# Factors Affecting the Mental Health and Wellbeing of Men in Nursing: A Systematic Review and Narrative Synthesis

**DOI:** 10.1111/ijn.70153

**Published:** 2026-05-10

**Authors:** Eric Lim, Mingxin Zhang, Kezia Higham, Frank Donnelly

**Affiliations:** ^1^ School of Nursing Murdoch University Perth Australia; ^2^ School of Health Medicine University of Sanya Sanya China; ^3^ North Metropolitan Tafe Perth Australia

**Keywords:** gender diversity, gender equality, literature review, male nurse, male nursing students, men in nursing, mental health, narrative synthesis, systematic review, wellbeing

## Abstract

**Background:**

The number of men entering nursing has steadily increased over recent decades. Male nurses contribute unique perspectives and skills; however, men working in female‐dominated occupations face distinct impacts on mental health and wellbeing.

**Research Aim:**

To identify factors that negatively impact the mental health and wellbeing of male nurses and students.

**Methods:**

This systematic literature review followed the five steps as outlined by Gregory and Denniss for conducting a systematic literature review and narrative synthesis.

**Results:**

Seventy research papers were included. Findings identified three major categories and eight factors describing the mental health and wellbeing of male nurses and students. Themes identified were ‘Social and Cultural Stigma’, ‘Institutional and Workplace Exclusion’ and ‘Hostility and Vulnerability in Practice’.

**Conclusions:**

Findings highlight systemic and interpersonal challenges to the mental health of male nurses and students, offering a foundation for future research, hypothesis generation and the development of targeted, evidence‐based strategies to address these issues.

## Summary

What Is Already Known About This Topic?

- Men working in female‐dominated professions, such as nursing, are at increased risk of poorer mental health and wellbeing.
- Existing literature acknowledges stigma and gender‐role conflict among male nurses but lacks a comprehensive synthesis of contributing factors.

What This Paper Adds?

- Findings identify eight interrelated factors that negatively affect the mental health and wellbeing of male nurses and nursing students.
- Offers a conceptual grouping of these factors into three overarching categories: *Social and Cultural Stigma*, *Institutional and Workplace Exclusion*, and *Hostility and Vulnerability in Practice*.

The Implications of This Paper

- Calls for urgent policy and educational reforms to foster gender inclusivity in nursing curricula and clinical environments.
- Highlights the need for targeted interventions such as mentorship programs, awareness campaigns, and structural changes to reduce stigma and discrimination.
- Provides an evidence base for future longitudinal and experimental research to develop and test strategies that improve retention and mental health outcomes for men in nursing.

## 1 Introduction and Background

Gender segregation of the workforce is a global phenomenon originating in the late nineteenth century when substantial numbers of women started to enter the workforce (Chan et al. 2014; Saleh et al. 2020). Gender segregation impacted the workforce as men and women were aligned to professions due to the normative work arrangements and types of appropriate tasks deemed appropriate for males and females (Milner et al. 2018). Consequently, women were expected to occupy caretaking and nurturing jobs, whereas men were expected to occupy authoritative, managerial and leadership roles (Froehlich et al. 2020). This led to a norm whereby female‐dominated jobs were often believed to merit lower pay and benefits than comparable male‐dominated jobs (Yavorsky and Dill 2020).

Gender segregation of the workforce has persisted across countries and over time (Froehlich et al. 2020; Milner et al. 2018). In today's twenty‐first century, men and women are often expected to occupy professions with certain traits in the global workforce (Froehlich et al. 2020; Milner et al. 2018). Nursing is a profession that has been recognised as a feminine job since Nightingale established the Nightingale Training School in 1860 (Chan et al. 2014; Saleh et al. 2020). At that time, Nightingale directed that nurses needed to be gentle, empathetic, caring and compassionate, traits typically ascribed to women (Ashkenazi et al. 2017). Despite employment campaigns to attract more men to nursing, it remains a predominantly female‐dominated profession (Froehlich et al. 2020; Saleh et al. 2020; Zhang and Liu 2016).

Nevertheless, increasing numbers of men are choosing to enter the nursing profession (Zhang and Liu 2016). In Australia, men comprise approximately 12% of the nursing workforce (Baker et al. 2023), which is a slight increase in numbers from 10.41% in 2014 (Stanley et al. 2016), a slow but increasing trend (Shudifat et al. 2023). A nurse who identifies their gender as ‘male’ is commonly referred to as a ‘male nurse’ as a ‘nurse’ is otherwise assumed to be female (Baker et al. 2023).

The literature indicates that male nurses may have a preference for certain areas of nursing practice. For example, it has been suggested that male nurses tend to be more technology savvy, thus may be more comfortable in areas of nursing with higher levels of technology such as critical care, emergency care and advanced practice settings (Petges and Sabio 2020; Saleh et al. 2020). Rabie suggests male nurses are thought to be physically stronger compared to their female colleagues, able to provide safer care for non‐ambulant patients and more able to move or assemble heavy equipment (Rabie et al. 2020). Most often, however, men who have ventured into a nursing career have encountered challenges impacting their mental health and wellbeing (Powers et al. 2018).

The challenges facing men in nursing may be considered in relation to two theoretical models. The social role theory (Eagly 1997) suggests that men are hard wired to look for and work in masculine vocations, such as construction, so that they could ‘learn’ about their gender role expectations (Wolfe and Levant 2023). Minority stress theory (Frost and Meyer 2023) suggests that as a marginal group, male nurses and students who are constantly faced with prejudice, stigma and discrimination will have a greater risk for negative mental health and wellbeing. Accordingly, to protect themselves from ill‐health, men may either abandon their decision to become a nurse or not see nursing as a lifelong career, reflected in high levels of attrition from the professions (Christensen et al. 2021; Feng et al. 2019; Feng et al. 2016; Gao et al. 2019; Liu et al. 2019; Popper‐Giveon et al. 2015; Saleh et al. 2020; Valizadeh et al. 2014). Such theories offer broad perspectives to be considered in understanding the complex task for policy makers and key stakeholders to implement mechanisms that can reduce the negative impact of minority stress for men in nursing (Frost and Meyer 2023). A recent systematic review and meta synthesis conducted by Wong et al. (2025) found that male nurses continued to be faced with gender inequality and discrimination within and beyond the workplace. The review by Wong et al. (2025) however did not explore the mental health consequences associated with the minority role that male nurses have. Male nurses and students continue to face challenges not experienced by female peers; thus, it is crucial for institutions and health services to identify ways to reduce the impacts of factors identified and discussed within this review to promote more positive lived experiences for male nurses and nursing students (Twidwell et al. 2022).

Evidence from an Australian national survey has shown that men employed in occupations where women are dominant have poorer mental health and wellbeing compared to men in male‐dominated occupations (Milner et al. 2018). For example, men who choose nursing as a career have faced stigma, discrimination, and negative work or academic experiences (Reedy et al. 2025). As such, male nurses and students are at a higher risk compared to their female counterparts of experiencing burnout, a loss of enthusiasm, transfer to another position or course, and expressing a desire to leave the nursing profession altogether (Reedy et al. 2025; Whitford et al. 2020). Although there are now more men choosing to be nurses, there is also a higher attrition rate for male nurses and students, meaning there is little overall progress in addressing the workforce imbalance (Twidwell et al. 2022). Despite this ongoing issue, there remains a lack of in‐depth understanding of the factors that negatively impact the mental health and wellbeing of male nurses and students. This knowledge is needed to inform targeted policy directives and public health education campaigns to reduce social and personal stigma toward men in nursing, and to support male nurses and students to experience positive mental health outcomes, work and academic experiences (Reedy et al. 2025).

### 1.1 Aim of the Study

The aim of this study was to identify factors that negatively impact the mental health and wellbeing of male nurses and students.

## 2 Methods

This systematic literature and narrative synthesis followed the five steps outlined by Gregory and Denniss (2018): (1) frame a research question, (2) search and re‐search the literature, (3) be critical, (4) find a logical structure and (5) write the narrative review. This systematic review was conducted and reported in accordance with the PRISMA 2020 guidelines (Moher et al. 2009).

### 2.1 Step 1—Frame a Research Question

The research question guiding this systematic review and narrative synthesis was: What factors negatively affect the mental health and wellbeing of men in nursing? The PICo mnemonic, which stands for Population, Phenomena of Interest, and the Context as described by the Joanna Briggs Institute (2014), guided the structuring of the research question and the search strategies. Table 1 presents the PICo mnemonic and the identified keywords.

**TABLE 1:** PICo mnemonic and the identified keywords.

PICo	Keywords
**Population**	Male nurse, men in nursing, male nursing student
**Phenomena of Interest**	Experiences, perceptions, attitudes, views, feelings, mental health
**Context**	Gender‐based interests

The inclusion criteria for review papers were literature related to the following: (i) male nurses, (ii) male nursing students and (iii) factors that could affect the mental health and wellbeing of male nurses. Studies were restricted to English language and primary and original research data. Literature reviews, opinion papers, conference proceedings and abstracts were omitted.

### 2.2 Step 2—Search and Re‐Search the Literature

A search for papers was conducted in January 2025 using the search strategy developed by a university librarian (see Table 2) to identify primary research papers reporting factors that can affect the mental health and wellbeing of men in nursing. The search for papers published from 2013 to date was conducted using the identified keywords (Scopus, Web of Science, ProQuest Central) and MESH terms (Medline, CINAHL). The decision to include articles in the last 12 years was guided by the increase in men who have entered the nursing profession during this period.

**TABLE 2:** Search and results.

	Concept 1	Concept 2	Results
**Keywords**	nurs* ADJ3 (male or man or men)	‘mental health’ or wellbeing or ‘well‐being’ or ‘self‐esteem’ or resilience or stereotyp* or stigma* or discriminat* or perception* or perceive* or masculine* or predjudic* or ‘career choice*’ or ‘lived experience*’	
**Subject headings**			
**Medline (Mesh)**	Nurses, Male/	Mental Health/	960
**CINAHL**	MH = ‘Nurses, Male’ or ‘Students, Nursing, Male’	MH = ‘Mental Health’	563
**Scopus**	Keywords only		483
**Web of Science**	Keywords only		6
**ProQuest Central**	Keywords only *Limit: Scholarly journals*		193

**FIGURE 1:**
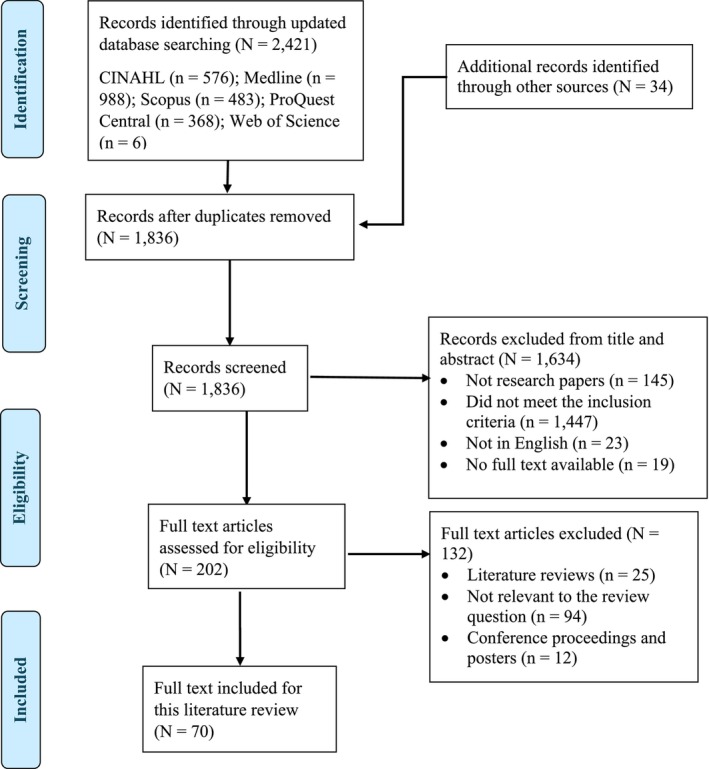
PRISMA flow diagram.

The process of conducting the review is detailed in Figure 1.

All relevant research papers published were identified, and the abstracts were downloaded. Title and abstracts were then screened by E.L. and K.H., and those that were not in English and those not meeting the inclusion criteria were removed. The search for papers generated a total of 2457 records; 619 duplicate records were removed. A further 1634 papers were omitted due to (i) not research papers (*n* = 145); (ii) did not meet the inclusion criteria (*n* = 1447); (iii) not in English (*n* = 23); and (iv) no full text available (*n* = 19). The full‐text papers of 201 records were downloaded, and eligibility assessment excluded an additional 131 records which were as follows: (i) literature reviews (*n* = 25); (ii) not relevant to the review question (*n* = 94); and (iii) conference proceedings and posters (*n* = 12). Finally, 70 papers were included for review. Appendix 1 is the quality assessment description of the final 70 papers completed by E.L. and K.H. using the Mixed Methods Appraisal Tool (MMAT (Hong et al. 2018).

### 2.3 Step 3—Be Critical

To ensure rigour the full texts of the included research papers were read and re‐read by E.L. and M.Z. to summarise the findings of the included papers that were relevant to the review and subsequently charted into Table 3. When reading and summarising, E.L. and M.Z. continuously reflected on the research question to check and validate the collected data. Headings used in Table 3 included the reference, country, method, participants, phenomenon of interest, brief overview of relevant findings, and identified factors. Following that, the tables completed by the two authors were compared (Barnett‐Page and Thomas 2009), and any disagreements that arose during the comparison were discussed with the K.H. until a consensus was achieved.

**TABLE 3:** Characteristics of included papers, brief overview of findings relevant to the review, and the identified factors.

No.	Author (year) /country	Method	Participants	Phenomenon of interest	Brief overview of findings relevant to the review	Identified factors
1*	Abushaikha et al. (2014) Jordan	Qualitative descriptive study using semi‐structured interviews	Male nursing students (*N* = 20)	Challenges and positive aspects that undergraduate male nursing students encounter during their study.	Male nursing students: Encountered daily negative stereotypes from other students. Experienced negative self‐image and resentment. Conveyed feelings of discrimination, anger, and dismay toward the admission and hospital policies that favour female nursing students. Expressed feelings of injustice toward limited future employment opportunities.	II, IV and V
2	Achora, S. (2016) Uganda	Qualitative descriptive study using in‐depth interviews	Male nurses (*N* = 11)	Experiences of men practicing nursing.	Persistent stereotype held by the communities that nurses are female. Being despised as people who have failed in life. Being accused by the other health care professionals as imposters wanting to dominate the nursing profession. Meeting resistance in clinical and healthcare settings where gender and age differences exist. Experiencing gender bias in the allocation assignment of complex nursing care. Being denied opportunities for career development and promotions.	I, II and V
3	Alboliteeh and Alshammari (2022) Saudi Arabia	Cross‐sectional survey study	Male nurses (*N* = 361)	Workforce influences that encourage male nurses to stay on job.	A lack of promotion opportunities as the main reason for leaving the profession.	V
4	Appiah et al. (2021) Ghana	Qualitative descriptive study using semi‐structured interviews	Male nurses (*N* = 20)	Experiences and motivation of male nurses in tertiary hospital.	Negative public perceptions about male nurses. Perceived as sexual predators Gender conflict when providing intimate care for female patients.	I and VI
5	Ayala et al. (2014) Chile	Qualitative study informed by gender socialisation theory (interviews)	Male and female beginner and advanced nursing students (*N* = 28)	Socialisation of male nursing students and masculine identity and the construction of inequalities in nursing education.	Family pressures causing men to discard the idea of nursing as a fitting career.	I
6	Baker et al. (2022) Australia	Qualitative descriptive interview study with nurses and patients	Male nurses (*N* = 23) and patients (*N* = 15)	Perceptions about male nurses.	Patients' ideology, gender stereotypes about nursing as women's work create a fear that men are sexual threats and unable to be trusted.	VI
7	Baker et al. (2023) Australia	Grounded theory study	Male nurses (*N* = 23) and patients (*N* = 15)	Perceptions about male nurses.	Male nurses felt added pressure to claim their personal and professional identity. Male nurses choose to work in urgent care environments over non‐urgent environments as there was a lesser risk of being misinterpreted by patients. Male nurses felt that they needed to purposefully develop a trusting relationship with their patients. Male nurses were constantly screening and scanning patients' verbal and non‐verbal interactions for risk of being misinterpreted.	V and VI
8	Baobaid et al. (2023) Malaysia	Cross‐sectional survey study	Male nurses (*N* = 88)	Experience of male nurses.	Engaging in traditionally female‐dominated roles prompted uncertainty about their masculinity. Promotion was an extrinsic reward but only for a minority. Extra‐visibility and stereotypes resulted in limitations on male nurses and added pressure, higher job strain, and higher levels of work stress which negatively impacts on overall performance.	I and V
9	Banakhar et al. (2021) Saudi Arabia	Qualitative descriptive study using semi‐structured interviews	Male nursing interns (*N* = 12)	Intention to study nursing.	There was a lack of public awareness of male nurses. There was a lack of clinical instructors for male nurses. There was a lack of practice opportunities for male nursing students during clinical placements. Religious and cultural beliefs challenged male nurses to care for female patients.	I, IV and VI
10	Blackley et al. (2019) Australia	Qualitative descriptive interview study	Male nurses (*N* = 6)	Stressors experienced by male nurses, strategies to cope with the stressors, and motivations to remain in the profession.	Disbeliefs from peers that nursing was their career choice. Male nurses felt excluded from workplace and social interactions. Male nurses experienced apprehension in providing personal care to female patients. Male nurses were disproportionately assigned ‘masculine’ tasks.	III, V, VI and VII
11	Carnevale and Priode (2018) America	Phenomenological qualitative study	Male nursing students (*N* = 11)	Lived experiences while enrolled in the undergraduate nursing program.	Disbelief from others that nursing was their career choice. Feeling excluded in clinical settings. Exposure to gender bias by nursing faculty and instructors.	III and IV
12	Carte and Williams (2017) America	Cross‐sectional correlational study	Male nurses (*N* = 37)	Relationship between the variables of demographics and causes of role strain among male registered nurses in critical care settings.	Male nurses experienced role ambiguity and role overload as the two most significant causes of role strain.	V
13	Chan and Fang (2023) China	Qualitative study using thematic analysis	Male nurses (*N* = 12)	How male nurses manage, negotiate, and reconstruct their masculinities in a feminised job.	Male nurses needed to negotiate entrepreneurial and breadwinner masculinities. Nursing was culturally devalued and regarded as a demeaning low‐skilled job. Male nurses experienced culturally specific stigma. Studying nursing was an embarrassment. Male nurses felt that they let their family down. Male nurses defended their masculinities by gravitating toward occupational spaces or specialities. Male nurses distanced themselves from general wards and female nursing colleagues.	I, II, III and V
14	Chan et al. (2014) Hong Kong	Qualitative descriptive study	Male nursing students (*N* = 18)	Positive and negative self‐image of male nursing students.	The assumption of nurses as female was common among patients and other health care staff. Common belief that male nurses were inferior to female nurses. Male nurses were perceived as less competent than female nurses when caring for female patients. Practice of male nurses and male students were limited by organisational barriers. Were concerned about the limited opportunities to refine nursing skills due to approaching patients of the opposite gender. Had concerns about putting themselves under suspicion if caring for female patients alone. Experienced being assigned extra tasks that increased their workload Treated as a ‘tool’ for carrying out certain tasks.	II, IV, VI and VII
15	Chang and Jeong (2021) South Korea	Qualitative study using thematic analysis	Male nurses (*N* = 10)	Gender discrimination and sexual harassment working as a nurse.	Institutional discrimination made male nurses think they could not stay. Burden of standing out as a man rather than a nurse. Experienced unfair treatment from nursing colleagues. Experienced sexual harassment at work as a man. Found it hard to be recognised as a sexual harassment victim.	V and VIII
16	Chen et al. (2020) China	Convergent mixed‐methods study	Male students (*N* = 237) and male nurses with < 3 years of experience (*N* = 33)	Professional identity of male nursing students and influencing factors of the professional identity.	Over half of the respondents held negative perceptions toward the nursing profession, pointing out nurses had lower social status and the job was laborious. Most respondents reported that they had been teased and discriminated against and were concerned that the stereotypical images may negatively impact their marriage prospects. 41% were reluctant to admit they were male nurses in front of new friends.	I and II
17	Cheng et al. (2018) Taiwan	Phenomenological qualitative study	Male nurses (*N* = 14)	The lived experiences of novice male nurses when they first enter the workplace.	Male nurses were caught in expectations of being the breadwinner. Male nurses assessed workplaces for the potential for professional growth. Male nurses faced challenging interpersonal relationships in the workplace. Male nurses experienced barriers to caring for young female patients.	I, V and VI
18	Chinkhata and Langley (2018) Malawi	Qualitative descriptive study	Male nursing students (*N* = 70)	Experience of male nurse midwives during undergraduate education.	Nursing activities were considered feminine. Male student nurse midwives were teased for having joined a feminine profession by either male students undertaking non‐nursing courses, or female nursing students, or qualified female nurse midwives. Male student nurse midwives felt inferior to men working in male dominated health professions. There was a sense of not belonging because of perceived discrimination from some of the patients, lecturers and qualified female student nurse midwives. Lack of male role models contributed to feelings of isolation in male student nurse midwives. Some female patients and/or their husbands refused to be cared for by either student or qualified male nurse midwives.	I, II, IV and VI
19	Christensen et al. (2018) Australia	Phenomenological qualitative study	Male nursing students (N = 8)	Lived experience of male nursing students.	Male nurses were met with a sense of quiet distaste or bewilderment from their family and friends. Male nurses experienced unexpected reactions from peers which made them feel angry, frustrated, and irritated at the innuendos made of them.	III
20	Christensen et al. (2021) Australia	Cross‐sectional survey study	Male nursing students (*N* = 14)	Male friendliness in nursing and gender role conflict.	Male nursing students were focused on climbing the career ladder to compensate for their lower self‐esteem. Male nursing students were faced with gender‐role conflict. Male nursing students were concerned that their touch and/or nursing care might be misconstrued by female patients as being sexual.	V and VI
21	Dos Santos (2022) South Korea	Qualitative study using thematic analysis	Male nursing students (*N* = 20)	Relationships between stress, workplace bullying, and experiences of male nursing students.	All participants shared negative experiences of mental distress due to their gender in the university environment. Almost all participants reconsidered their career pathways in nursing due to negative experiences in nursing college. Male nursing students experienced negative experiences and biases from their patients, family, friends and public. There were no male professors, male role models, and male patients in student‐run clinics. Many observed unfairness and bias from their female counterparts in the hospital. Male nursing students were expected to do all the labour and responsibilities while female counterparts did almost nothing.	I, II, IV, V and VII
22	Gao et al. (2019) China	Qualitative descriptive study	Male nursing students (*N* = 14)	Educational experience of nursing.	Male nursing students faced gender stereotyping, a lack of male role models, social isolation, and teaching strategies a aimed toward female learners. Male nursing students might not pursue a lifelong career as the social status of nursing is not high. Male nursing students felt uncertain about whether they should continue in the nursing profession after graduation.	I, II and IV
23	George and Bhatti (2019) India	Qualitative descriptive study	Male nurses (*N* = 20)	Reasons why male nurses select the profession and their gendered experiences during their career paths.	Nursing is not a suitable profession for men. Male nurses experienced stereotyping and marginalisation in public. Male nurses received negative treatment from their family and friends. Male nurses found it difficult to perform their duties because of taunts from their patients.	I, III and VI
24	Göktepe and Sarıköse (2022) South Korea	Qualitative descriptive study	Male nurses (*N* = 10)	Male nurses' experiences of workplace gender discrimination and sexual harassment.	Faced gender discrimination at work from various dimensions. Experienced unfair treatment from female nursing colleagues. Burden of standing out as a ‘man’ rather than as a ‘nurse’ Institutional discrimination that made male nurses think they cannot stay longer. Experienced subtle forms of sexual harassment at work and we are not recognised as a victim.	V and VIII
25	Guy et al. (2022) New Zealand	Qualitative descriptive study	Male nurses (*N* = 9)	Factors that impact why men do not view nursing as a career choice.	Nursing was not prominent in their awareness when leaving school and making career choices. Men in nursing experienced isolation due to the societal gendering of nursing influencing the participant's knowledge and understandings of what nursing was, as a career.	I and II
26	Harding et al. (2018) New Zealand	Qualitative descriptive study	Male nursing students (N = 8)	Reasons for men's enrolment in the first three intakes of the inaugural New Zealand second degree graduate entry (SDGE) nursing course.	Majority of the male nurses experienced difficulties getting married. Majority felt embarrassed to tell people about their job and had lied about their job at least once to public. Most of male nurses believed that there was a lack of career growth and hiring limitations due to their gender. Some felt that their manliness was either questioned, or they were seen as predators.	I, II, V and VI
27	Hosseini et al. (2022) Iran	Cross‐sectional questionnaire survey	Male nursing students (*N* = 128)	Male nursing students' perception of gender barriers in nursing curricula.	There were five main educational barriers: Faculty's referral to nurses using feminine prepositions. Different treatments against male and female nursing students. Different requirements/limitations in obstetric (OB) apprenticeship. Needing to prove oneself because of people's expectation of nurses to be female. Getting nervous after being accused of sexual inappropriateness after touching female patients.	IV, V and VI
28	Huang et al. (2024) China	Qualitative descriptive study	Male nursing students (*N* = 11) and male nursing professionals (*N* = 11)	Feelings, thoughts, and opinions of male nursing students and male nursing professionals about their perception of the nursing profession.	Male nurses faced pressure and challenges at nursing schools and health institutions as a minority. Male students reported feeling ignored during group work. Male nurses faced limited employment prospects in interventional departments. Male nurses pursued higher education and/or career advancement due to internal and external pressures Male nurses experienced care refusal by female patients.	IV, V and VI
29	Jeong and Chang (2022) South Korea	Cross‐sectional survey study	Male nurses (*N* = 155)	Factors that influenced sexual harassment experience of male nurses.	Majority (65.2%) of male nurses had experienced sexual harassment at least once at work.	VIII
30	Kalemba (2020) South Africa	Qualitative study informed by gender and masculinity theory	Male nurses (*N* = 15)	Gender performative practices through which black male nurses negotiate their masculinity.	Male nurses experienced being accused of having subordinate gender identities including being gay. Male nurses experienced being called ‘sister’ by patients and other nurses. Male nurses experienced marginalisation when there was a lack of toilets for men. Male nurses experienced tense relationships with their female colleagues. Male nurses were excused from completing tasks which the patients associated with a feminine role.	I, II and V
31	Kluczyńska (2017) Poland	Qualitative descriptive study	Male nurses (*N* = 17)	Main motives for choosing nursing and for leaving the profession.	Nursing as a feminised profession caused a delay in some of the participants' occupational choice. Men usually resigned from nursing because of low income or reluctance to perform tasks of lower prestige, defined as feminine and a negative atmosphere at work.	I
32	Liu and Li (2017) Taiwan	Qualitative descriptive study	Male nursing students (*N* = 10); female nursing students (*N* = 12)	The gendered experiences of male nursing students during their first initial nursing clinical practice.	Male nursing students needed to constantly de‐construct and re‐construct their masculinity. Male nursing students were perceived as requiring more attention, encouragement, and support from academic and clinical teachers. Gender stereotypes shaped male nurses' behaviours. Male nurses might display traits of masculinity by setting their future career goals as head nurses, nurse practitioners, or managerial positions. Male nursing students experienced occupational gender segregation and rejection by patients and other co‐workers. Male nurses might experience embarrassment or nervousness when providing physical care to female patients.	IV, V and VI
33	Lyu et al. (2024) China	Grounded theory study	Male nurses (*N* = 25)	Male nurses' experiences of providing intimate care to female patients.	Male nurses were often apprehensive about using physical touch and feared being accused of inappropriate use of touch. Male nurses' had low self‐confidence in providing intimate care. Male nurses were concerned with traditional influences and with the provision of intimate care to female patients.	VI
34	Mao et al. (2021) China	Qualitative descriptive study	Male nursing students (*N* = 12); male nurses (*N* = 12)	The advantages and disadvantages of being a male in the nursing profession.	As a minority, male nurses experienced pressure and difficulties in nursing school and health institutes. Male nursing students were marginalised in group tasks and practice. Male nurses were restricted in the choice of employment. Some female patients denied male nurses' care in private parts. Male nurses were perceived as physically strong and good at operating machines.	IV, V, VI and VII
35	Martínez‐Morato et al. (2021) Spain	Qualitative study using thematic analysis	Male nurses (*N* = 12)	Male nurses' descriptions of emotion management and the impact of gender stereotypes.	Nursing was often seen as a career for women and that male nurses faced misconceptions when it came to emotional care.	I
36	Maryunani et al. (2021) Indonesia	Phenomenological qualitative study	Male nurses (*N* = 12)	The experience of male nurses caring for female patients.	Both female patients and male nurses experienced a feeling of discomfort.	VI
37	Mohammadi et al. (2020) Iran	Qualitative descriptive study	Males nursing students (*N* = 12)	Male students' understanding of the concept of dignity.	The lack of support could lead to low self‐worth and even resignation.	IV
38	Ndou and Moloko‐Phiri (2018) South Africa	Qualitative descriptive study	Males nursing students (*N* = 30–40)	Male students' experiences in a profession traditionally perceived as a female domain.	Terms like ‘sister’ reinforced gender stereotypes, causing discomfort. Male students felt excluded in wards dominated by female nurses and peers, leading to feelings of not belonging and lacking male role models and mentors. Male students were often assigned physically demanding tasks (e.g., lifting patients, cleaning) instead of clinical skills practice. Female students were prioritised for workshops and hands‐on tasks, while males were sidelined except for ‘manpower’ roles.	II, IV and VII
39	O'Connor (2015) Ireland	Qualitative narrative study	Males nursing students (*N* = 18)	Gender‐related issues men face when choosing a career in nursing, and how they negotiate male identity in this female‐dominated profession.	Men faced gender identity dilemmas when choosing a career in nursing, and their choices are often questioned by family, friends, and society. Participants distanced themselves from feminine stereotypes of nursing (e.g., nurturing, emotional labour) to align with hegemonic masculinity (e.g., breadwinning, rationality). Male nurses were often perceived as homosexual, which negatively affects career advancement. Male students faced assumptions about being homosexual or ‘effeminate’, which they dismissed as outdated stereotypes. The lack of male role models and career guidance in the nursing profession made it difficult for men to advance their careers.	I, II and IV
40	Petges and Sabio (2020) America	Qualitative descriptive study	Male nursing students (*N* = 13)	Reason for choosing nursing and the lived experience of men in the nursing course.	Nursing was not a good career choice for men. Men in nursing were considered lower in social status. Male nurses faced social constraints within their community i.e., refusal of their daughters to marry male nurses. Male nurses felt embarrassed with being associated with a female role.	I and II
41	Popper‐Giveon et al. (2015) Israel	Mixed	Assessment of data from the Labour Force Survey Male nurses (*N* = 7); female nurses (*N* = 6)	Percentage of men in nursing and their career choice.	Male nurses preferred to work in nursing roles that are associated with hegemonic masculinity (physical strengths, stressful environment, having special professional authority, and heavy responsibility). Male nurses purposefully distanced themselves from nursing and femininity.	V
42	Powers et al. (2018) America	Qualitative descriptive study	Former enrolled male nursing students (*N* = 11)	Lived experience of former male nursing students.	Gender bias existed in class. Former enrolled male nursing students experienced feeling excluded or being treated differently than female students when seeking additional assistance in the form of advice, examination review, or remediation. Male nursing students felt they were singled out, both negatively and positively, in class or in clinical settings because they were male. There was a lack of exposure to male faculty members and male nurses Male nurses were excluded from care provision in certain settings due to being male. Male nurses were frequently asked to assist in lifting heavy patients or to provide care to violent patients.	IV, V and VII
43	Þorsteinsdóttir & Gislason (2024) Iceland	Qualitative comparative study	Male nurses (*N* = 7); female nurses (*N* = 10)	The impact of gender stereotypes on nurses' career choices and jobs.	Male nurses entering the nursing profession needed to overcome the barriers of traditional perceptions. Male nurses were often questioned by the outside world. Male nurses faced the problems caused by gender stereotypes in their work.	I and II
44	Prosen (2022) Slovenia	Qualitative descriptive study	Male nursing students (*N* = 14); female nursing students (*N* = 58)	Nursing students' perceptions of gender‐defining roles in nursing and future career roles.	Most students believed that the nursing curriculum reflects gender equality to some extent, but others believe that the curriculum is more focused on the female perspective. Male students occasionally experienced gender differences in teaching and practice, such as the lack of attention paid to male students by clinical instructors. Male students were more focused on management, leadership, and technical aspects of their future career roles and see themselves as having an edge in these areas. Patients were more receptive to male nurses, but in some cases, such as the gynaecology ward, patients may feel embarrassed. Male nurses were more desirable team members because they have more physical strength work ethic.	IV, V, VI and VII
45	Rabie et al. (2020) South Africa	Qualitative descriptive study	Male nurses (*N* = 10); female nurses (*N* = 10); patients (*N* = 10)	Stereotypes of occupational roles about male nurses.	Male nurses experienced stereotyping and influences of stereotypes on work performance.	V
46	Salamonson et al. (2023) Australia	Cross‐sectional comparative survey study	Male nursing students (*N* = 641); female nursing students (*N* = 587)	Nursing students' perceptions of whether men nursing students were treated differently during clinical placement.	Male nursing students felt (a) bullied, outcasted; (b) judged poorly; (c) frozen out, no support; (d) exploited, (e) used as ‘muscles’ (f) not fit for nursing; and (g) fewer opportunities during their clinical placement.	IV, V and VII
47	Saleh et al. (2020) Jordan	Phenomenological qualitative study	Male nurses (*N* = 22)	Experiences of their career within their Arabic community.	Male nurses faced social constraints within the community such as people refused to marry their daughters to male nurses. Nursing was stereotyped as a female‐dominated career. Male nurses felt disgraced that their children see them at work because of their image in the family. Male nurses were not welcome to perform some procedures. Male nurses were not welcome in the obstetrics, gynaecology, and paediatric sections due to social norm dominance. Male nurses preferred not to provide direct bedside care to stay behind the scenes. Male nurses expected themselves to do the heavy lifting.	I, II, III, V and VII
48	Salvador and Mohammed Alanazi (2024) Saudi Arabia	Qualitative descriptive study	Male nurses (*N* = 23)	Stereotypes and challenges faced by male nurses in the nursing profession, as well as perceptions of the industry's development.	The lack of male role models in the nursing profession made it difficult for male nurses to advance their careers. Male nurses faced sexism and discrimination in the workplace which led to issues such as workplace violence and horizontal bullying. Male nurses felt unsatisfied in a female‐dominated profession. Despite the advantages of male nurses in some ways, there was a conflict between their male identities and nursing roles.	IV and V
49	Sayman (2015) America	Qualitative descriptive study	Male nurses (*N* = 10)	The experiences and challenges of male working in nursing.	Most of the participants did not initially choose a career in nursing as they felt isolated and stereotyped in nursing school. In nursing schools and workplaces, male nurses faced negative stereotypes and verbal harassment from doctors and patients, feeling that their male identity did not match the nursing profession, and marginalisation. Half of the participants left the nursing profession, were dissatisfied with their jobs, and felt limited in their career development, such as not being able to be promoted, being seen as a workhorse Gender stereotypes led male nurses to many obstacles in their careers, such as lower salaries, fewer rewards, and lower professional status. Male nurses were often treated with disrespect, rudeness, and experienced problems with relationships with colleagues and doctors.	II and V
50	Sedgwick and Kellett (2015) Canada	Cross‐sectional survey study	Male nursing students (*N* = 33); female nursing students (*N* = 429)	Differences in the sense of belonging between male and female undergraduate nursing students in clinical practice.	Factors such as a female‐dominated culture in the nursing profession, gender role stereotypes, unequal opportunities for career advancement, and male students' own gender identity contributed to male students' lower sense of belonging in some areas. Male students were reluctant to ask for help or advice from colleagues in clinical practice environments. Male students felt less capable and confident in their nursing practice.	I, II and IV
51	Sevilla and Rangel (2024) Chile	Cross‐sectional survey study	Male nursing students (*N* = 209); female nursing students (*N* = 443)	The impact of gender differences on the career development of male nurses.	Male students received less parental support. Male students perceived more barriers than women in their career development, but these barriers did not affect their self‐efficacy and career aspirations.	III and IV
52	Shakwane (2022) South Africa	Qualitative descriptive study	Male nursing students (*N* = 12)	The experiences and problems faced by male nursing students in providing intimate care.	Traditional gender role stereotypes were reinforced in nursing practice, limiting diversity and innovation in care. Misconceptions faced by male nursing students could lead them to experience self‐doubt and confusion, affecting the quality of care. Male nursing students viewed intimate care such as basic physical needs and touch as important means of achieving nursing goals. Touching the patient's body was taboo in some cultures, which creates confusion for male nursing students when providing intimate care.	IV and VI
53	Silva‐Sánchez et al. (2024) Chile	Qualitative descriptive study	Male nursing students (*N* = 15)	Male nursing students' perception of undergraduate training.	Gender stereotypes influenced students' choice of nursing careers, with some students believing that nursing is a women's profession and that men's choice of nursing is questioned. Teachers' traditional attitudes led to different expectations of male and female students, affecting their learning experience and achievement. Males were expected to have greater physical strength and leadership skills.	II, IV and VII
54	Shudifat et al. (2023) Jordan	Qualitative descriptive study	Male nurses (*N* = 28)	Factors that encourage or discourage male nursing students from pursuing a career in nursing.	Gender stereotypes affected the male nursing students' desire to remain in nursing. Male nursing students avoided talking about their career due to humiliation about doing a job that was perceived as female job.	II
55	Smith and Horne (2024) America	Qualitative descriptive study	Male nurses (*N* = 11)	Male nurses' experiences in modern nursing environment.	Male nursing teachers felt isolated in their teaching, and that the female‐dominated groups of teachers may ignore or belittle their opinions. The lack of male mentors and leaders affected the career development of male nurses. The lack of attention and support for male nurses in nursing education led to a lack of knowledge and skills among male nurses. There was gender discrimination in recruitment and work arrangements, and male nurses were often assigned to specific patient groups, such as obese patients or patients who need to be carried.	IV and VII
56	Smith et al. (2020) America	Qualitative descriptive study	Male nurses (*N* = 11)	Lived experiences of male nurses.	Male nurses experienced negative societal views that devalue male nurses. Male nurses needed to adapt to the clinical and academic environment to avoid isolation and to ensure professional survival. Male nurses experienced stereotypical attitudes from other health professionals. Male nurses were limited by their gender to provide intimate female care. Male nurses faced rejection by female patients and their families.	I, IV, V and VI
57	Stanley et al. (2016) Australia	Cross‐sectional survey study	Male nurses (*N* = 247); female nurses (*N* = 808)	Perception of men in nursing.	Negative stereotype of men in nursing. Being seen as ‘muscle’ by female colleagues.	II and VII
58	Subu et al. (2023) The United Arab Emirates	Qualitative descriptive study	Male nurses (*N* = 30)	Barriers and facilitators to male choice of nursing profession.	The image and social perception of the nursing profession had an important impact on the choice of male students Traditional gender stereotypes and prejudices led to barriers for males to choose a nursing profession.	I and II
59	Subu et al. (2022) The United Arab Emirates	Qualitative descriptive study	Male nurses (*N* = 30)	Male nursing students' perceptions of the nursing profession and reasons for choosing a career in nursing.	There was a general perception in society that nursing is a profession for women, and that men are subject to discrimination and prejudice when pursuing nursing work, which makes it very stressful for men to choose a nursing profession. Gender stereotypes in the nursing profession were one of the major challenges faced by male nursing students, leading to social discrimination and prejudice against male nursing students, affecting their career progression. Some parents believed that nursing jobs are not suitable for men and oppose their children's choice of nursing professions.	I, II and III
60	Turan et al. (2021) Turkey	Qualitative descriptive study	Male nurses (*N* = 17); female nurses (*N* = 7)	Feelings, thoughts and opinions of nursing students about gender perception in nursing.	The low status of the nursing profession and the negative image of gender equality have led to a higher turnover rate among male nurses. Religious beliefs, family upbringing, and culture had an impact on students' gender perceptions, resulting in males facing challenges in the nursing profession. Male nurses faced societal stereotypes and discrimination in the nursing profession, such as when working in female‐dominated departments such as obstetrics and gynaecology.	I, II and V
61	Twomey and Meadus (2016) Canada	Cross‐sectional survey study	Male nurses (*N* = 240)	Reasons for male nurses to choose a career in nursing, barriers they face, recruitment and retention strategies, and career satisfaction.	The lack of images of male nurses in the media was a hindrance to men's choice of nursing professions. The top barrier was being seen as muscle, and men felt that they were always asked to do the heavy work.	I and VII
62	Valizadeh et al. (2014) Iran	Qualitative descriptive study	Male nurses (*N* = 18)	Public view of nurses, and their perceptions of male nurses.	Public viewed nursing as a feminine and physician‐subordinate career. Male nurses experienced the embarrassment of revealing their careers as a nurse.	I and II
63	Vatandost et al. (2020) Iran	Phenomenological qualitative study	Male nurses (*N* = 20)	Challenges faced by male nurses in caring for female patients.	Organisations required male nurses to provide care, but when patients or their relatives object, male nurses are not supported and may be punished, which leads to job stress and unfair treatment of male nurses. Male nurses were religiously and culturally restricted when performing some nursing procedures that involve female patient privacy. Patients or their relatives tended to refuse care from male nurses. Male nurses feared being misconstrued. Most male nurses had experienced other challenges in providing care to female patients that had limited their ability to offer their full professional capabilities.	V and VI
64	Whitford et al. (2020) Scotland	Sequential explanatory mixed‐methods study	Male nursing students (*N* = 33); university and college nursing lecturers (*N* = 21); school teachers (*N* = 46)	Influences and causes of the underrepresentation of men in pre‐registration nursing.	Nursing was not a job that men should be doing. The perceived low earning potential of nursing could be challenging for men as they are seen as the ‘bread winner’. The assumption of femininity associated with the profession challenged the masculine identity of male students. Difficulty for male nurses to provide intimate care to female patients.	I, II and VI
65	Xian et al. (2020) China	Cross‐sectional correlational study	Male nurses (*N* = 366)	Burnout and job demand.	Male nurses experienced cultural and social stressors Younger male nurses with lower monthly salaries were more prone to burnout. Male nurses had a strong need for career advancement and tend to be working in high‐intensity conditions. Male nurses had high job demands i.e., longer working days or shifts. Male nurses received lower work resources i.e., social support, reward, and skill diversity.	I, V and VII
66	Yada et al. (2014) Japan	Cross‐sectional correlational study	Male nurses (*N* = 85); female nurses (*N* = 159)	Job‐related stress.	Male nurses experienced stronger gender consciousness than females resulting in anxiety and poorer attitude toward nursing. Male nurses were usually required to deal with aggressive psychiatric patients. Male nurses were usually tasked with inappropriate demands resulting in self‐doubt, lowered self‐esteem, depressed moods, and reduced confidence in their ability to care for patients.	VI and VII
67	Yang et al. (2017) Taiwan	Qualitative descriptive study	Male nursing students (*N* = 24)	Nursing learning experiences and coping strategies.	In social life, there were curiosity, doubts, and misconceptions about male nursing students, such as suspicion of their sexual orientation. Male students faced barriers in their learning environment and clinical practice. Some instructors lowered their expectations of the male nursing students believing they were less likely than the female nursing students to become a nurse. Gynaecological and obstetric patients, often rejected the male nursing students.	II,IV and IV
68	Yokoya et al. (2023) Japan	Qualitative descriptive study	Male nursing students (*N* = 20)	Barriers and coping strategies in nursing education.	Traditional gender stereotypes influenced the career choices of male students. Traditionally, gender roles had been associated with female traits, and male students may feel confused and uneasy about their professional roles. The emphasis on female nurses and the use of traditional terminology in the textbook reinforced the notion that nursing is a female profession, confusing male students. In group activities and practical teaching, male students tended to receive more attention and differential treatment, which leads to increased learning pressure. Male nursing students experienced rejection from patients in their maternal and infant care practices, which leaves them frustrated and confused and may affect their professional confidence.	II, IV and VI
69	Yip et al. (2021) Hong Kong	Qualitative descriptive study	Male nursing students (*N* = 22)	Gender‐related perceptions of male nursing students in clinical placement.	Nursing schools were gender‐neutral in their teaching, but there is a bias against male nursing students in clinical settings for practical reasons to protect students and avoid complaints. Participants felt that there was a need for more male role models in clinical settings, which helped to elevate the status of male nurses and provide support and guidance to male nursing students. Male nursing students felt alienated in the obstetrics and gynaecology practicum and often treated differently by nurses, excluded from important practice areas, and felt like visitors rather than students, an experience that made them feel hurt. Male nursing students were expected to take on heavy physical tasks because they were physically strong, which led to an uneven distribution of learning opportunities and affected their participation in important learning activities and practical activities.	IV, V and VII
70	Zhou et al. (2024) China	Qualitative descriptive study	Male nursing students (*N* = 7)	Clinical internship experiences of male nursing students.	Male nursing students hoped that the hospital would pay attention to gender differences and provide appropriate facilities and environments. Male nursing students believed that there was a gap between the theories learned in school and clinical practice, and more practice was needed. The preparation before the internship appeared to be insufficient in clinical practice, and patients had doubts about the abilities of male nursing students. The participants said that they experienced various difficulties and challenges in clinical practice related to not only inadequate preparation for internships but also psychological burnout. Male nursing students had advantages in physical strength and flexibility, but their theoretical knowledge is relatively weak. They are willing to take on physical work and work hard to overcome their weaknesses. During the male nursing students' clinical internship, they took on many other roles than being students.	IV and VII

*Note:* Factor I—see papers numbered 2, 4, 5, 8, 9, 13, 16, 17, 18, 21, 22, 23, 25, 26, 30, 31, 35, 39, 40, 43, 47, 50, 56, 58, 59, 60, 61, 62, 64 and 65. Factor II—see papers numbered 1, 2, 13, 14, 16, 18, 21, 22, 25, 26, 30, 38, 39, 40, 43, 47, 49, 50, 53, 54, 57, 58, 59, 60, 62, 64, 67 and 68. Factor III—see papers numbered 10, 11, 13, 19, 23, 47, 51 and 59. Factor IV—see papers numbered 1, 9, 11, 14, 18, 21, 22, 27, 28, 32, 34, 37, 38, 39, 42, 44, 46, 48, 50, 51, 52, 53, 55, 56, 67, 68, 69 and 70. Factor V—see papers numbered 1, 2, 3, 7, 8, 10, 12, 13, 15, 17, 20, 21, 24, 26, 27, 28, 30, 32, 34, 41, 42, 44, 45, 46, 47, 48, 49, 56, 60, 63, 65 and 69. Factor VI—see papers numbered 10, 14, 21, 34, 38, 42, 44, 46, 47, 53, 55, 57, 61, 65, 66, 69 and 70. Factor VII—see papers numbered 4, 6, 7, 9, 10, 14, 17, 18, 20, 26, 27, 28, 32, 33, 34, 36, 44, 56, 63, 64, 66, 67 and 68. Factor VIII—see papers numbered 15, 24 and 29.

### 2.4 Steps 4 and 5—Find a Logical Structure and Writing the Review

The constant comparative method of analysis (Charmaz 2006) was used in the narrative synthesis of the collected data. E.L. and M.Z. read and re‐read the brief overview of findings relevant to the review independently to identify the codes and themes. Identified codes and themes were then compared and grouped into their relevant categories (Barnett‐Page and Thomas 2009). Following this, the codes and themes of each of the categories were read and re‐read by E.L. to allow their meanings to emerge and shape the direction of the narrative synthesis (Barnett‐Page and Thomas 2009). Finally, the narrative synthesis of the categories and factors were reviewed and checked by all the members of the research team for consensus, and this step enhanced the trustworthiness and credibility of the process of data analysing, narrative synthesising and writing up of the findings (Johnson et al. 2020).

## 3 Results

The 70 included papers originated from China (*n* = 8), United States (*n* = 7), Australia (*n* = 7), Iran (*n* = 4), South Korea (*n* = 4), Chile (*n* = 3), Jordan (*n* = 3), Saudi Arabia (*n* = 3), Taiwan (*n* = 3), Canada (*n* = 2), Hong Kong (*n* = 2), Japan (*n* = 2), New Zealand (*n* = 2), South Africa (*n* = 4), The United Arab Emirates (*n* = 2), and one each from the following countries in alphabetical order: Ghana, Iceland, India, Indonesia, Ireland, Israel, Malaysia, Malawi, Poland, Scotland, Slovenia, Spain, Turkey and Uganda.

### 3.1 Findings

The focus of our systematic review and narrative synthesis was identifying factors that negatively affect the mental health of men in nursing. However, during analysis, it was identified that there were some factors that contribute positively to the mental health of male nurses. For example, individuals who experienced nursing as a source of economic stability, job variety, and career advancement including global opportunities, reported a sense of personal resonance and satisfaction about their choice of becoming a nurse (Chen et al. 2020; Christensen et al. 2021; Gao et al. 2019; Harding et al. 2018; Kluczyńska 2017; Mao et al. 2020; O'Connor 2015; Popper‐Giveon et al. 2015; Saleh et al. 2020; Shudifat et al. 2023; Smith et al. 2022; Twomey and Meadus 2016; Yang et al. 2017; Zhang and Tu 2020). Individuals who felt that they were helping others and contributing to society also reported a sense of altruism and self‐fulfilment about working as a nurse (Abushaikha et al. 2014; Appiah et al. 2021; Christensen et al. 2018; Harding et al. 2018; Kluczyńska 2017; Shudifat et al. 2023; Twomey and Meadus 2016; Yang et al. 2017). Despite these observations, most studies identified that male nurses suffer more negative mental health challenges than they gain.

**FIGURE 2:**
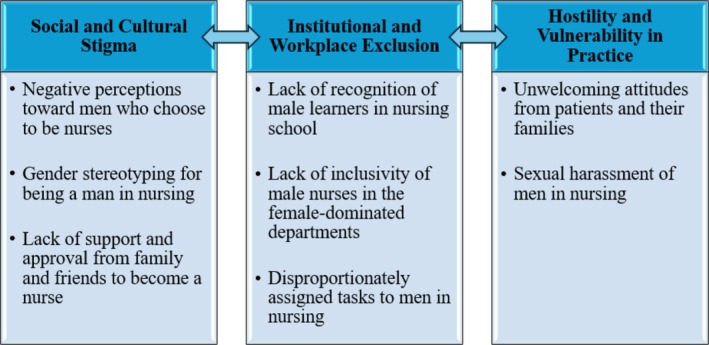
Major categories of the identified factors and their potential to interrelate or reinforce each other.

Subsequently, the analysis identified eight factors impacting the mental health and wellbeing of men in nursing. These factors were as follows: (I) negative perceptions toward men who chose to be a nurse, (II) gender stereotyping for being a man in nursing, (III) lack of support and approval from family and friends to become a nurse, (IV) lack of recognition of male learners in nursing schools, (V) lack of inclusivity of male nurses in female‐dominated departments, (VI) tasks disproportionately assigned to men in nursing, (VII) unwelcoming attitudes from patients and their families and (VIII) sexual harassment of men in nursing. The eight identified factors were further compared and grouped into three major categories, which interrelate and reinforce each other (see Figure 2).

#### 3.1.1 Factor I—Negative Perceptions Toward Men Who Chose to Be a Nurse

Contemporaneously, nursing continues to be regarded as feminine in nature and is still broadly perceived as a profession meant only for women or for individuals who considered their gender as female (Achora 2016; Maram Banakhar et al. 2021; Chinkhata and Langley 2018; George and Bhatti 2019; Guy et al. 2022; Martínez‐Morato et al. 2021; Sedgwick and Kellett 2015; Subu et al. 2022; Twomey and Meadus 2016; Valizadeh et al. 2014; Whitford et al. 2020; Xian et al. 2020). As such, men who choose to be nurses are often faced with direct or indirect questions about their career choice (O'Connor 2015; Þorsteinsdóttir and Gíslason 2024), their gender identity (Kalemba 2020), masculinity (Baobaid et al. 2023) and their ability to raise a family (Xian et al. 2020). Those who were faced with disapprovals from their family, friends or others could also develop self‐doubt and uncertainty about their decision to become a nurse (Ayala et al. 2014; Baobaid et al. 2023; Harding et al. 2018; Kluczyńska 2017; Smith et al. 2022; Subu et al. 2023; Turan et al. 2021).

It was consistently noted that female oriented professions were poorly paid and had lower status compared to male hegemonic societies where it is considered the role of men to be the primary bread winner of their families (Chan and Fang 2023; Chen et al. 2020; O'Connor 2015; Þorsteinsdóttir and Gíslason 2024; Whitford et al. 2020). Consequently, men who work in female preponderance professions were perceived as having lower socioeconomic status and personal achievements (Achora 2016; Chan and Fang 2023; Cheng et al. 2018; George and Bhatti 2019; Petges and Sabio 2020; Whitford et al. 2020). In some conservative societies, men who do not work in male preponderance professions were even faced with marriage refusals (Harding et al. 2018; Petges and Sabio 2020; Saleh et al. 2020). All these negative experiences could be significant in making male nurses and students feel inferior to other men who choose to work in male preponderance professions (Chinkhata et al. 2018).

#### 3.1.2 Factor II—Gender Stereotyping for Being a Man in Nursing

It was reported that most male nurses and students have had their gender questioned, teased or were discriminated against for joining a feminine profession (Chen et al. 2020; Chinkhata et al. 2018; O'Connor 2015; Silva‐Sánchez et al. 2024; Yang et al. 2017). For male students, it was reported that they had been ridiculed by non‐nursing male students, female nursing students and even qualified female nurses (Abushaikha et al. 2014; Chinkhata et al. 2018; Dos Santos 2022; Whitford et al. 2020). Their experience of gender stereotyping because of their career choice could result in feelings of embarrassment (Chan and Fang 2023; Petges and Sabio 2020; Valizadeh et al. 2014), humiliation (Shudifat et al. 2023), discrimination (Subu et al. 2022), confusion and being uneasy (Yokoya et al. 2023), all of which could potentially impact on their mental health and wellbeing. It was reported that some male students who developed negative self‐image and resentment (Abushaikha et al. 2014), had lied about their career choice (Chen et al. 2024; Harding et al. 2018), had socially isolated themselves (Gao et al. 2023; Guy et al. 2022) or changed their decision to become a nurse (Gao et al. 2019; Sayman 2015; Shudifat et al. 2023; Silva‐Sánchez et al. 2024; Subu et al. 2023; Subu et al. 2022; Turan et al. 2021; Yokoya et al. 2023).

For some male students, gender stereotyping could persist even after they became nurses (Stanley et al. 2016; Þorsteinsdóttir and Gíslason 2024). For instance, the terms used to refer to nurses, such as ‘sister’ in the clinical setting may serve as a constant reminder that nursing is a female‐dominated career (Kalemba 2020; Ndou and Moloko‐Phiri 2018; Saleh et al. 2020; Sedgwick and Kellett 2015). It was reported that some patients and healthcare professionals saw male nurses as inferior when compared to female nurses due to the limitations imposed by their gender (Chan et al. 2014) or as imposters wanting to dominate the nursing profession in the clinical settings (Achora 2016).

#### 3.1.3 Factor III—Lack of Support and Approval From Family and Friends to Become a Nurse

Evidence suggested that men were more likely faced with disbeliefs, negative attitudes, a sense of quiet distaste, or bewilderments from their family and friends (Carnevale and Priode 2018; Martin Christensen et al. 2018; Sevilla and Rangel 2024; Subu et al. 2022). The unexpected reactions from family and friends could affect their mental health and wellbeing if they experienced anger, frustration and irritation at the innuendos made of them (Christensen et al. 2018). For some, the lack of encouragement from family and friends could lead them to develop low self‐esteem or self‐worth (Chan and Fang 2023), and this could escalate to the feeling of shame and disgrace for letting their family down (Saleh et al. 2020).

#### 3.1.4 Factor IV—Lack of Recognition of Male Learners in Nursing Schools

Male students experienced a significant gender bias in the nursing curriculum (Prosen 2022; Silva‐Sánchez et al. 2024; Yip et al. 2021). It was reported that feminine prepositions were still used by the faculty members and in the textbook and clinical practice environment to refer to nurses or nursing students (Carnevale and Priode 2018; Hosseini et al. 2022; Yokoya et al. 2023). As such, male students felt that they would need to constantly adapt to the feminine clinical and academic environment if they want to become nurses (Liu and Li 2017; Smith et al. 2020).

Additionally, there was a lack of male academics to role model and guide male learners, or the use of diverse and innovative teaching strategies effective for teaching male learners to overcome the challenges of being a male nurse (Banakhar et al. 2021; Chinkhata et al. 2018; Dos Santos 2022; Gao et al. 2019; O'Connor 2015; Powers et al. 2018; Salvador and Mohammed Alanazi 2024; Shakwane 2022; Smith et al. 2024; Yip et al. 2021). The use of a gender‐biased nursing curriculum may make male learners feel that they do not belong to this profession, that they are bullied or that they are outcasts. For some, the treatment from faculty members could cause them to develop negative feelings of discrimination, anger, and dismay toward the nursing profession (Abushaikha et al. 2014; Ndou and Moloko‐Phiri 2018; Salamonson et al. 2023) and ultimately to reconsider their career pathways (Dos Santos 2022).

Moreover, male students also experienced a lack of practice opportunities during clinical placements (Banakhar et al. 2021; Salamonson et al. 2023; Yang et al. 2017). This issue was frequently related to organisational barriers (Chan et al. 2014; Sevilla and Rangel 2024; Zhou et al. 2024) or practical reasons such as to protect them from getting complaints (Chinkhata et al. 2018; Shakwane 2022; Yip et al. 2021). The lack of clinical practice opportunities may lead nurses and patients to perceive that male students are inadequately prepared compared to their female counterparts to provide patient care independently (Chan et al. 2014; Zhou et al. 2024). As such, male students could be ignored, excluded, belittled, or treated differently by their clinical instructors, nurses, other health professionals and patients (Carnevale and Priode 2018; Chinkhata et al. 2018; Hosseini et al. 2022; Huang et al. 2024; Mao et al. 2021; Powers et al. 2018; Prosen 2022; Sedgwick and Kellett 2015; Smith and Horne 2024; Yang et al. 2017; Yokoya et al. 2023). Consequently, some male students may develop low self‐confidence and self‐doubt about their clinical skills and knowledge to work as nurses after graduation (Liu and Li 2017; Mao et al. 2021; Mohammadi et al. 2020; Sedgwick and Kellett 2015; Smith et al. 2024; Zhou et al. 2024).

#### 3.1.5 Factor V—Lack of Inclusivity of Male Nurses in Female‐Dominated Departments

It was reported that there was an absence of toilets for men in some female‐dominated departments such as obstetrics, gynaecology and paediatrics, making male nurses feel unwelcomed, discriminated, alienated or stereotyped because of their gender (Chang and Jeong 2021; Göktepe and Sarıköse 2022; Hosseini et al. 2022; Kalemba 2020; Saleh et al. 2020; Turan et al. 2021; Yip et al. 2021). In these female‐dominated departments, male nurses often felt that they were excluded from opportunities to perform complex nursing care, experienced unfair treatment from female nursing colleagues, or were met with resistance to demonstrate their skills and knowledge because of gender bias or social norm dominance (Achora 2016; Blackley et al. 2019; Chan et al. 2014; Chang and Jeong 2021; Dos Santos 2022; Göktepe and Sarıköse 2022; Kalemba 2020; Powers et al. 2018; Saleh et al. 2020). In some cases, the sexism and discrimination toward male nurses could escalate to workplace violence and horizontal bullying (Salvador and Mohammed Alanazi 2024). For instance, it was reported that male nurses experienced verbal harassment, disrespect, rudeness and problems with relationships with colleagues, doctors and patients who felt that their male identity did not match the nursing profession (Sayman 2015; Smith et al. 2020). As such, they find it challenging to establish positive collegial relationships leading to feeling alienated, lonely and isolated in the workplace (Blackley et al. 2019; Cheng et al. 2018; Kalemba 2020).

Furthermore, there was a perceived lack of clarity of their roles for male nurses working in female‐dominated departments (Carte and Williams 2017). This situation leads male nurses to experience role strain, stress and job dissatisfaction (Carte and Williams 2017; Salvador and Mohammed Alanazi 2024; Sayman 2015). As such, many male nurses were likely to distance themselves from female‐dominated departments (Chan and Fang 2023; Mao et al. 2021), even if their decision could impact on their career trajectories (Liu and Li 2017). Even if a male nurse decides to work in a female‐dominated department, they could still face resistance for career development and promotion due to their lack of opportunities to perform clinically and ultimately burnout (Achora 2016; Alboliteeh and Alshammari 2022; Baobaid et al. 2023; Harding et al. 2018; Huang et al. 2024; Sayman 2015; Smith et al. 2020; Vatandost et al. 2020).

Consequently, most male nurses are likely to work in departments that are dominated by male nurses such as mental health units to avoid the burden of standing out in other clinical settings (Cheng et al. 2018; Göktepe and Sarıköse 2022). Ironically to avoid the stress of being a nurse essentially compounding the stress they already suffer (Baker et al. 2023; Baobaid et al. 2023; Hosseini et al. 2022; Popper‐Giveon et al. 2015; Rabie et al. 2020). For some male nurses, the pursuit of higher education qualifications, working as head nurses or nurse practitioners or moving into management, leadership and specialist roles, was regarded as a means to protect or maintain their mental health and wellbeing (Christensen et al. 2021; Huang 2024; Liu et al. 2024; Prosen 2022; Saleh et al. 2020; Xian et al. 2020).

#### 3.1.6 Factor VI—Tasks Disproportionately Assigned to Men in Nursing

There was a general assumption among the men that because of their gender, they ought to be ‘masculine’ and therefore should do ‘physically demanding’ tasks such as lifting and cleaning of bedridden patients, or managing the behaviour of violent and aggressive patients (Blackley et al. 2019; Ndou and Moloko‐Phiri 2018; Mao et al. 2021; Powers et al. 2018; Prosen 2022; Silva‐Sánchez et al. 2024; Smith and Horne 2024; Stanley et al. 2016; Yada et al. 2014). Therefore, male nurses and students tend to be assigned to more physical roles over caring roles, thus contributing to uneven distributions of practice opportunities and longer working days or shifts (Ndou and Moloko‐Phiri 2018; Xian et al. 2020; Yip et al. 2021; Zhou et al. 2024). For some, they were required to assume extra physical tasks on top of their regular clinical workload (Chan et al. 2014). The lack of practice opportunities hinders the knowledge and skills advancement of male nurses and students and reduces their competence and confidence to care for patients (Twomey and Meadus 2016; Yada et al. 2014). Subsequently, many male nurses and students learned to accept that they would need to do the physical work, receive lower work resources when performing physical tasks, essentially being exploited for choosing to work in a female‐dominated profession (Chan et al. 2014; Dos Santos 2022; Salamonson et al. 2023; Saleh et al. 2020; Stanley et al. 2016; Xian et al. 2020). These negative attitudes could instil self‐doubt, lowered self‐esteem, depressed moods, and reduced satisfaction and impact on their mental health and wellbeing (Dos Santos 2022; Salamonson et al. 2023).

#### 3.1.7 Factor VII—Unwelcoming Attitudes From Patients and Their Families

Negative experience resulted from unwelcome attitudes and gender conflict due to the prevailing ideology and stereotype about nursing being a women's profession (Appiah et al. 2021; Baker et al. 2022; Christensen et al. 2018). In many situations, male nurses and students caring for female patients are challenged by social, religious and cultural beliefs and practices (Banakhar et al. 2021; Cheng et al. 2018; Lyu et al. 2024; Shakwane 2022; Vatandost et al. 2020). Almost all male nurses and students had experienced refusal of care by patients and their families (Smith et al. 2020). Male nurses and students who experienced this reported feeling frustrated, confused, rejected, with reduced confidence to provide clinical care and treatment (Chinkhata et al. 2018; Huang et al. 2024; Liu and Li 2017; Mao et al. 2021; Smith et al. 2020; Yang et al. 2017; Yokoya et al. 2023). In some instances, patients and their families could even display attitudes or behaviours that hinted that male nurses and students were sexual threats or sexual predators (Appiah et al. 2021; Baker et al. 2022; Chan et al. 2014; Christensen et al. 2021; George and Bhatti 2019; Harding et al. 2018; Hosseini et al. 2022). This was especially so if they were providing care to the person's private or intimate areas of the body (Christensen et al. 2021; Lyu et al. 2022; Maryunani et al. 2021; Prosen 2022; Vatandost et al. 2020; Whitford et al. 2020).

To care for a person who displayed unwelcoming attitudes, many male nurses and students would purposefully develop a trusting relationship, constantly screen and scan for verbal and non‐verbal cues and signs of being misinterpreted (Baker et al. 2023). However, the detection of any signs of distrust and discomfort expressed by the patient would often cause the male nurse or student to experience anxiety, embarrassment or nervousness, all of which could affect their personal mental health and wellbeing (Blackley et al. 2019; Liu and Li 2017; Lyu et al. 2024; Vatandost et al. 2020; Yada et al. 2014).

#### 3.1.8 Factor VIII—Sexual Harassment Experienced by Men in Nursing

Three papers contributed to Factor VIII and revealed the sexual harassment experienced by men in nursing. While this phenomenon is not well‐researched, most male nurses reported having experienced sexual harassment at least once at work (Jeong and Chang 2022). However, most male nurses and students believed that it was difficult for others to see them as sexual harassment victims, as the incidents were either often too subtle or ‘serious’ enough to be objectively reported due to their gender orientation (Chang and Jeong 2021; Göktepe and Sarıköse 2022).

## 4 Discussion

This systematic review has identified several themes highlighting that male nurses face significant personal and professional cultural and stereotypical barriers. These barriers can generate a sense of rejection, loneliness and a lack of professional acceptance, which can then be the precursors of deeper mental health concerns (Akinyemi et al. 2025). At a time when the global shortage of nurses is predicted to worsen, the profession must provide structural and cultural change to welcome and sustain men into nursing. As such, the factors noted within this study offer a comprehensive set of findings that can drive interventions for policy makers and key stakeholders to identify targeted supportive strategies and initiatives to support male nurses and nursing students to enable a more positive workplace and institutional experience and reduce attrition rates of men who choose to be nurses (Kluczyńska 2017; Reedy et al. 2025).

All around the world, there is now an urgent call to address the gender imbalance that has long existed in the nursing workforce to reflect the demographic of the public it serves (Froehlich et al. 2020; Milner et al. 2018; Nerges et al. 2022). Emerging evidence highlights the importance of having diversity in the nursing workforce to provide high quality patient care, achieve more culturally appropriate care, and to increase the effectiveness of care and treatment (Berdida 2023; Nerges et al. 2022). Male nurses are regarded as valuable members of the nursing teams in the fields of mental health, critical care, emergency care, and the operating theatre and military nursing (Rabie et al. 2020; Smallheer et al. 2020). They are also seen as valuable members of nursing teams providing trauma‐informed care for men who have experienced past and present trauma or gender‐sensitive care for men with certain religious obligations in regard to having physical contact with the opposite sex (Budu et al. 2019; McKinnish et al.; Rabie et al. 2020).

It is also important to note that the quality of nursing care is closely connected with the mental wellbeing of the nursing workforce (Babapour et al. 2022). Understanding that some proportion of the workforce, men, may have mental health concerns acknowledges that this group of people may not be providing the highest level of care expected. It is critical that nursing as a profession owns the quality of care provided from any cohort within and works tirelessly to improve health care outcomes. Tacitly accepting that a proportion of a professional workforce is at risk of additional stress due to their gender reduces the profession.

Changing social norms and structures is acknowledged to be a very difficult task (Kiptulon et al. 2024). Moving the perception of a discipline among people where societal perspectives are deeply ingrained is a challenging but necessary step forward if the nursing profession is to see a significant and sustained balance of gender. Curiously, the role of a male obstetrician is accepted far more readily than a male midwife and yet both roles working primarily with pregnant women have uneven levels of social and professional acceptance.

### 4.1 Recommendations

Firstly, our findings highlighted the importance of ensuring gender diversity and equality in nursing education to support male nurses and students to develop their professional identity and a sense of belongingness (Fitzgerald 2020; Powers et al. 2018). This may include ‘rebranding’ nursing as a gender‐neutral profession at the governmental and political level to acknowledge the increasingly growing numbers in male students in recent years (Jordal and Heggen 2015). This may result in the use of more gender‐neutral language and pictorial illustrations in textbooks and teaching materials (Bell‐Scriber 2008). In addition, education on the history of the nursing profession should include the history of men in nursing and their contributions to the profession (Anthony 2004; Bell‐Scriber 2008). This may significantly increase appreciation and awareness for male nurses and students, thus reducing negative stereotyping from female students of other academic majors (Banakhar et al. 2021).

Secondly, our findings highlighted the important role that institutions could have to employ more male nurse educators. Nursing schools, with predominantly more female educators, reflect the workplace environment, essentially highlighting that the gender divide transcends clinical environments (Kulakac et al. 2015; Sayman 2015). As such, teaching and learning environments may not take into consideration the unique learning needs and differing communication styles of male students (Anthony 2004; O'Lynn 2003). Consequently, if there are more gender‐balanced nurse educators, male students can be provided with gender‐based activities and interactions to learn about the role of male nurses in a variety of healthcare settings, how to care for patients, and to be accepted into the nursing profession (Banakhar et al. 2021; Jordal and Heggen 2015; Petges and Sabio 2020). Male academics in the nursing faculty can act as role models, sharing their experiences in nursing and helping male students develop their professional identity (Anthony 2004; Jordal and Heggen 2015; Sayman 2015). Furthermore, female nurse educators and students can also be more prepared and comfortable to work with male nurses and students (Kulakac et al. 2015; Sayman 2015).

Thirdly, our findings highlighted the importance of recognising and addressing the workplace incivility that male nurses and students face in the clinical environment. Male nurses and students were found to have greater psychosocial hazards due to the low representation of men among the nursing workforce (Kowalczuk et al. 2018). For example, awareness education or campaigns could be based on the evidence of this paper, such as the identified categories and factors, to increase awareness of the mental health and wellbeing of male nurses and students (Kowalczuk et al. 2018; Reedy et al. 2025). Targeted clinical supervision and mentoring programs could be provided for male nurses and students to support them to develop their professional identity, socialise with others to avoid feelings of isolation, and to ensure professional survival (Liu et al. 2019; Smith et al. 2020).

Lastly, our findings highlighted the need to conduct longitudinal research to obtain an in‐depth understanding of experiences that could negatively impact the mental health and wellbeing of men in nursing. There is also a need to conduct experimental research to test strategies and interventions intended to promote gender equality in the nursing profession. This research should take into consideration the intersectionality of men, for example their cultural differences, and the impact of ethnicity and religion.

### 4.2 Limitations

This systematic literature review and narrative synthesis is not without limitations. Firstly, we limited the search for papers from 2013 to date and might not have included relevant papers that are outside this range. Nevertheless, the range was determined by the research team to capture contemporary issues that could negatively impact the mental health and wellbeing of men in nursing. Secondly, we only included papers written in English. However, we were able to identify 70 papers to conduct this systematic review and narrative synthesis and identified three major categories with eight factors to provide an in‐depth understanding of experiences that could negatively impact on the mental health and wellbeing of men in nursing. Lastly, we did not compare the intersectionality of men but acknowledged this may mitigate the effects of the identified categories and factors in our systematic review and narrative synthesis.

## 5 Conclusion

The findings of this systematic literature review and narrative synthesis provide evidence that men in nursing, like others working in professions with female preponderance, are potentially challenged by factors that can threaten their mental health and wellbeing. As such, it is important for institutions and health services to create awareness and address the factors that could contribute to the psychosocial hazards faced by male nurses and students. The findings of our study can be used to develop hypotheses for testing using experimental research methods, generating empirical evidence for strategies and interventions to foster inclusivity and positive experiences for male nurses and students. The mental health and wellbeing of existing and future generations of male nurses depends on making positive change.

## Author Contributions

**Eric Lim:** conceptualization, formal analysis, data curation, writing ‐ original draft, supervision. **Mingxin Zhang:** conceptualization, formal analysis, data curation, writing ‐ review & editing. **Kezia Higham:** formal analysis, data curation, writing ‐ review & editing. **Frank Donnelly:** supervision, writing ‐ review & editing.

## Funding

The authors have nothing to report.

## Disclosure

The authors declare no conflicts of interest.

## Data Availability

The data that support the findings of this study are available from the corresponding author upon reasonable request.

## Appendix

**Table:**

			Quantitative (non‐randomised)					Qualitative				
	Are there clear research questions?	Do the collected data allow to address the research questions?	Are the participants representative of the target population?	Are the measurements appropriate regarding both outcome and intervention?	Are there complete outcome data?	Are the confounders accounted for in the design and analysis?	During the study period, is the intervention administered as intended?	Is the qualitative approach appropriate to answer the research questions?	Are the qualitative data collection methods adequate to address the research question?	Are the findings adequately derived from the data?	Is the interpretation of results sufficiently substantiated by data?	Is there coherence between qualitative data sources, collection, analysis, and interpretation?
**Abushaikha et al. (** 2014 **)**	▲	▲	n/a	n/a	n/a	n/a	n/a	▲	▲	▲	▲	▲
**Achora (** 2016 **)**	▲	▲	n/a	n/a	n/a	n/a	n/a	▲	▲	▲	▲	▲
**Adeyemi‐Adelanwa et al. (** 2016 **)**	▲	▲	▲	▲	▲	n/a	n/a	n/a	n/a	n/a	n/a	n/a
**Alboliteeh and Alshammari** (2022)	▲	▲	▲	▲	▲	n/a	n/a	n/a	n/a	n/a	n/a	n/a
**Appiah et al. (** 2021 **)**	▲	▲	n/a	n/a	n/a	n/a	n/a	▲	▲	▲	▲	▲
**Ayala et al. (** 2014 **)**	▲	▲	n/a	n/a	n/a	n/a	n/a	▲	▲	▲	▲	▲
**Baker et al. (** 2022 **)**	▲	▲	n/a	n/a	n/a	n/a	n/a	▲	▲	▲	▲	▲
**Baker et al. (** 2023 **)**	▲	▲	n/a	n/a	n/a	n/a	n/a	▲	▲	▲	▲	▲
**Baobaid et al. (** 2023 **)**	▲	▲	▲	▲	▲	n/a	n/a	n/a	n/a	n/a	n/a	n/a
**Banakhar et al. (** 2021 **)**	▲	▲	n/a	n/a	n/a	n/a	n/a	▲	▲	▲	▲	▲
**Blackley et al. (** 2019 **)**	▲	▲	n/a	n/a	n/a	n/a	n/a	▲	▲	▲	▲	▲
**Carnevale and Priode** (2018)	▲	▲	n/a	n/a	n/a	n/a	n/a	▲	▲	▲	▲	▲
**Carte and Williams** (2017)	▲	▲	▲	▲	▲	n/a	n/a	n/a	n/a	n/a	n/a	n/a
**Chan and Fang** (2023)	▲	▲	n/a	n/a	n/a	n/a	n/a	▲	▲	▲	▲	▲
**Chan et al. (** 2014 **)**	▲	▲	n/a	n/a	n/a	n/a	n/a	▲	▲	▲	▲	▲
**Chang and Jeong** (2021)	▲	▲	n/a	n/a	n/a	n/a	n/a	▲	▲	▲	▲	▲
**Chen et al. (** 2020 **)**	▲	▲	▲	▲	▲	n/a	n/a	n/a	n/a	n/a	n/a	n/a
**Cheng et al. (** 2018 **)**	▲	▲	n/a	n/a	n/a	n/a	n/a	▲	▲	▲	▲	▲
**Chinkhata and Langley** (2018)	▲	▲	n/a	n/a	n/a	n/a	n/a	▲	▲	▲	▲	▲
**Christensen et al. (** 2018 **)**	▲	▲	n/a	n/a	n/a	n/a	n/a	▲	▲	▲	▲	▲
**Christensen et al. (** 2021 **)**	▲	▲	▲	▲	▲	n/a	n/a	n/a	n/a	n/a	n/a	n/a
**Dos Santos (** 2022 **)**	▲	▲	n/a	n/a	n/a	n/a	n/a	▲	▲	▲	▲	▲
**Gao et al. (** 2019 **)**	▲	▲	n/a	n/a	n/a	n/a	n/a	▲	▲	▲	▲	▲
**George and Bhatti** (2019)	▲	▲	n/a	n/a	n/a	n/a	n/a	▲	▲	▲	▲	▲
**Guy et al. (** 2022 **)**	▲	▲	n/a	n/a	n/a	n/a	n/a	▲	▲	▲	▲	▲
**Harding et al. (** 2018 **)**	▲	▲	n/a	n/a	n/a	n/a	n/a	▲	▲	▲	▲	▲
**Hosseini et al. (** 2022 **)**	▲	▲	▲	▲	▲	n/a	n/a	n/a	n/a	n/a	n/a	n/a
**Huang et al. (** 2024 **)**	▲	▲	n/a	n/a	n/a	n/a	n/a	▲	▲	▲	▲	▲
**Jeong and Chang** (2022)	▲	▲	▲	▲	▲	n/a	n/a	n/a	n/a	n/a	n/a	n/a
**Kalemba (** 2020 **)**	▲	▲	n/a	n/a	n/a	n/a	n/a	▲	▲	▲	▲	▲
**Kluczyńska** (2017)	▲	▲	n/a	n/a	n/a	n/a	n/a	▲	▲	▲	▲	▲
**Liu and Li** (2017)	▲	▲	n/a	n/a	n/a	n/a	n/a	▲	▲	▲	▲	▲
**Lyu et al. (** 2024 **)**	▲	▲	n/a	n/a	n/a	n/a	n/a	▲	▲	▲	▲	▲
**Mao et al. (** 2021 **)**	▲	▲	n/a	n/a	n/a	n/a	n/a	▲	▲	▲	▲	▲
**Martínez‐Morato et al. (** 2021 **)**	▲	▲	n/a	n/a	n/a	n/a	n/a	▲	▲	▲	▲	▲
**Maryunani et al. (** 2021 **)**	▲	▲	n/a	n/a	n/a	n/a	n/a	▲	▲	▲	▲	▲
**Mohammadi et al. (** 2020 **)**	▲	▲	n/a	n/a	n/a	n/a	n/a	▲	▲	▲	▲	▲
**Ndou and Moloko‐Phiri** (2018)	▲	▲	n/a	n/a	n/a	n/a	n/a	▲	▲	▲	▲	▲
**O'Connor (** 2015 **)**	▲	▲	n/a	n/a	n/a	n/a	n/a	▲	▲	▲	▲	▲
**Petges and Sabio** (2020)	▲	▲	n/a	n/a	n/a	n/a	n/a	▲	▲	▲	▲	▲
**Popper‐Giveon et al. (** 2015 **)**	▲	▲	▲	▲	▲	—	n/a	▲	▲	▲	▲	▲
**Powers et al. (** 2018 **)**	▲	▲	n/a	n/a	n/a	n/a	n/a	▲	▲	▲	▲	▲
**Þorsteinsdóttir and Gislason (** 2024 **)**	▲	▲	n/a	n/a	n/a	n/a	n/a	▲	▲	▲	▲	▲
**Prosen (** 2022 **)**	▲	▲	n/a	n/a	n/a	n/a	n/a	▲	▲	▲	▲	▲
**Rabie et al. (** 2020 **)**	▲	▲	n/a	n/a	n/a	n/a	n/a	▲	▲	▲	▲	▲
**Salamonson et al. (** 2023 **)**	▲	▲	▲	▲	▲	n/a	n/a	n/a	n/a	n/a	n/a	n/a
**Saleh et al. (** 2020 **)**	▲	▲	n/a	n/a	n/a	n/a	n/a	▲	▲	▲	▲	▲
**Salvador and Mohammed Alanazi** (2024)	▲	▲	n/a	n/a	n/a	n/a	n/a	▲	▲	▲	▲	▲
**Sayman (** 2015 **)**	▲	▲	n/a	n/a	n/a	n/a	n/a	▲	▲	▲	▲	▲
**Sedgwick and Kellet (** 2015 **)**	▲	▲	▲	▲	▲	n/a	n/a	n/a	n/a	n/a	n/a	n/a
**Sevilla and Rangel** (2024)	▲	▲	▲	▲	▲	n/a	n/a	n/a	n/a	n/a	n/a	n/a
**Shakwane (** 2022 **)**	▲	▲	n/a	n/a	n/a	n/a	n/a	▲	▲	▲	▲	▲
**Silva‐Sánchez et al. (** 2024 **)**	▲	▲	n/a	n/a	n/a	n/a	n/a	▲	▲	▲	▲	▲
**Shudifat et al.** (2023)	▲	▲	n/a	n/a	n/a	n/a	n/a	▲	▲	▲	▲	▲
**Smith and Horne** (2024)	▲	▲	n/a	n/a	n/a	n/a	n/a	▲	▲	▲	▲	▲
**Smith et al. (** 2022 **)**	▲	▲	n/a	n/a	n/a	n/a	n/a	▲	▲	▲	▲	▲
**Smith et al. (** 2020 **)**	▲	▲	n/a	n/a	n/a	n/a	n/a	▲	▲	▲	▲	▲
**Stanley et al. (** 2016 **)**	▲	▲	▲	▲	▲	n/a	n/a	n/a	n/a	n/a	n/a	n/a
**Subu et al. (** 2023 **)**	▲	▲	n/a	n/a	n/a	n/a	n/a	▲	▲	▲	▲	▲
**Subu et al. (** 2022 **)**	▲	▲	n/a	n/a	n/a	n/a	n/a	▲	▲	▲	▲	▲
**Turan et al. (** 2021 **)**	▲	▲	n/a	n/a	n/a	n/a	n/a	▲	▲	▲	▲	▲
**Twomey and Meadus** (2016)	▲	▲	▲	▲	▲	n/a	n/a	n/a	n/a	n/a	n/a	n/a
**Valizadeh et al. (** 2014 **)**	▲	▲	n/a	n/a	n/a	n/a	n/a	▲	▲	▲	▲	▲
**Vatandost et al. (** 2020 **)**	▲	▲	n/a	n/a	n/a	n/a	n/a	▲	▲	▲	▲	▲
**Whitford et al. (** 2020 **)**	▲	—	▲	—	—	n/a	n/a	▲	▲	▲	▲	▲
**Xian et al. (** 2020 **)**	▲	▲	▲	▲	▲	n/a	n/a	n/a	n/a	n/a	n/a	n/a
**Yada et al. (** 2014 **)**	▲	▲	▲	▲	▲	n/a	n/a	n/a	n/a	n/a	n/a	n/a
**Yang et al. (** 2017 **)**	▲	▲	n/a	n/a	n/a	n/a	n/a	▲	▲	▲	▲	▲
**Yokoya et al. (** 2023 **)**	▲	▲	n/a	n/a	n/a	n/a	n/a	▲	▲	▲	▲	▲
**Yip et al. (** 2021 **)**	▲	▲	n/a	n/a	n/a	n/a	n/a	▲	▲	▲	▲	▲
**Zhang and Tu** (2020)	▲	▲	n/a	n/a	n/a	n/a	n/a	▲	▲	▲	▲	▲
**Zhou et al. (** 2024 **)**	▲	▲	n/a	n/a	n/a	n/a	n/a	▲	▲	▲	▲	▲

*Note:* Black triangle ▲: The criterion was assessed as being met. Inversed grey triangle ▼: The criterion was not met. Grey dash —: Unclear whether this criterion was met.

Abbreviations: n/a, not applicable.
